# Conducting Violence Research Across Multiple Family Generations and with Young Children: Findings from a Mixed-Methods Pilot Study in South Africa

**DOI:** 10.1007/s42448-023-00157-w

**Published:** 2023-03-07

**Authors:** Hannabeth Franchino-Olsen, Nicola Christofides, Nataly Woollett, Ansie Fouche, Mpho Silima, Christina Thurston, Kopano Monaisa, Franziska Meinck

**Affiliations:** 1 School of Social and Political Sciences, University of Edinburgh, Edinburgh, UK; 2 School of Public Health, University of the Witwatersrand, Johannesburg, South Africa; 3 Department of Visual Arts, University of Johannesburg, Johannesburg, South Africa; 4 School of Health Sciences, North-West University, Vanderbijlpark, South Africa; 5 Department of Social Wellbeing, United Arab Emirates University, A1 Ain, United Arab Emirates

**Keywords:** Child abuse, Violence, Intergenerational transmission, Young children

## Abstract

This paper presents findings from a pilot study focused on examining intergenerational violence in a three-generation sample, which included young children, in a rural area of South Africa. The aims of the pilot study were to investigate the feasibility of participant recruitment, consent, and interviewing; length and burden of the study questionnaires; appropriateness and acceptability of the measures used; and young children’s (age 4–7) ability to comprehend the measures and participate meaningfully in interviews asking about violence. Data were collected for 4 months with three groups of participants, often within families (young adults, their children, and the young adults’ former caregivers), using cognitive interviews, quantitative questionnaires, and qualitative in-depth interviews. All groups participated in arts-based methods and child interviews included visual and tactile aids. Pilot study findings demonstrated feasible recruitment within families for a three-generation study using comprehensive consent protocols and mandatory reporting information. Adults and young children were able to participate in the extensive interviews (2–3 h and 1 h, respectively) without significant burden. The employed measures were appropriate and acceptable to the setting, though minor revisions were made to improve comprehension of certain items. Young children were able to engage and participate meaningfully in the research, though they were not able to answer abstract reasoning items in cognitive interviews and children who were less developmentally advanced required more play- and arts-based accommodations to support their participation. Future research around sensitive topics, such as violence, appears feasible within families and including young children as participants even in resource-poor settings.

**Supplementary Information:**

The online version contains supplementary material available at 10.1007/s42448-023-00157-w.

## Introduction

Violence, particularly violence against children, is a major concern and societal burden, as it impacts health across the life course and can influence younger generations by affecting parenting and discipline practices (Assink et al., 2018; UNICEF, 2014; World Health Organization, 2016; World Health Organization, 2022). Research on violence is difficult to conduct due the sensitive and stigmatised nature of the topic (ISPCAN, 2016; Neelakantan et al., 2022). It is especially difficult to measure violence in an ethical manner across generations within families and to include young children in research about violence (Devries et al., 2015; Ellsberg & Heise, 2005; ISPCAN, 2016; Jewkes et al., 2012). As a result, there is very little research investigating violence which collects data from children under age 10, including studies seeking to collect multi-generational violence data within a family (Bradbury-Jones & Taylor, 2015; Powell & Smith, 2009; Schneider et al., 2015). Participation of young children has been demonstrated as feasible and argued for in previous work, such as through the use of forensic or investigative interviews (Hershkowitz et al., 2012; Katz et al., 2012, 2018) and questionnaires with close- and open-ended questions (Montserrat & Casas, 2017), and researchers have employed rights-based arguments to advocate for greater inclusion of young children in studies investigating their experiences of violence and abuse (Bruck et al., 2022; Kosher & Ben-Arieh, 2020). Exceptions to the gap in intergenerational data with young children include the three-generation Birth to Thirty cohort in South Africa (Richter et al., 2018; von Fintel & Richter, 2019; L. Richter, personal communication, 25 October 2022) and the three-generation Oregon Youth Study (Capaldi et al., 2018). These longitudinal data cohorts have been used to examine the nature and patterns of violence from infancy and early childhood to adolescence (Richter et al., 2018) as well as the intergenerational transmission of abuse and physical maltreatment (Capaldi et al., 2019; Pears & Capaldi, 2001), thus expanding our understanding the intergenerational links for violence and other health outcomes.

Despite these valuable intergenerational insights from the Birth to Thirty and Oregon Youth Study cohorts, the drivers and mechanisms for the intergenerational transmission of violence are poorly understood and represent a major research gap (Assink et al., 2018; Widom & Wilson, 2015; Widom et al., 2015). The Interrupt_Violence study (currently underway; 2022–2024) will seek to fill this knowledge gap by collecting data in a three-generation, longitudinal study in Mpumalanga, a resource-poor province of South Africa. The Interrupt_Violence main study is aimed at following up an existing longitudinal cohort of 1665 young adults (originally interviewed as adolescents in 2010–2011 and in 2011–2012) while also recruiting their oldest child and their former primary caregiver. More details on the methodology used in the study can be found elsewhere. In preparation for the longitudinal Interrupt_Violence study and to satisfy ethical review requirements, an in-depth, cross-sectional pilot study was conducted from July to October 2021 which recruited three generations of participants within a family not originally included in the Interrupt_Violence cohort.

This paper describes the elements and aims of the pilot study, highlighting key findings for collecting violence data from young children and across familial generations. The primary aims of the pilot study were to:

1. Establish the feasibility of recruiting participants, especially young children, into a study on intergenerational violence, gaining consent/assent within and across family generations (young adult consent to participate; parental/young adult consent for children’s participation; child assent to participate; young adult consent to contact their former caregiver; caregiver consent to participate), and interviewing participants in an ethical manner that appropriately protects them from harm.
2. Establish the length of the study questionnaires and their burden on the three groups of study participants (children, young adults, and former caregivers).
3. Examine the appropriateness of the measures employed in the study ranging from validated scales and items developed for the specific context to art-based activities, with a focus on violence items.
4. Explore the comprehension of the measures for children aged 4–7 years and their ability to participate in quantitative and qualitative research on violence.

## Methods

### Pilot Study Design

In preparation for a larger, longitudinal cohort study, a pilot study was conducted in rural Mpumalanga province of South Africa in July–October 2021. The pilot study consisted of a cross-sectional sample drawn from the residents living in two villages not originally sampled in the Interrupt_Violence study. Approval for the study was sought and granted from the chief (regional head), indunas (village head), and ward councillors (politically elected representatives) prior to study recruitment and data collection, as well as the ethics committees at the associated universities. Participants and locations of this pilot study did not overlap with participants in the main study which this pilot aimed to inform but were suitably similar—living in the same communities and speaking the same local language—to draw conclusions from this study.

### Staff and Training

Pilot study staff consisted of six local fieldworkers, a project manager, and a social worker. All fieldworkers were proficient in XiTsonga (the local language), had a minimum of a high school education, had prior fieldwork experience, and were hired and trained specifically for the pilot study. They underwent a multi-week, in-depth, in-person training course which employed experiential learning, critical reflection, group work, and direct instruction focused on (a) gender norms, (b) violence against women and children, (c) child development, (d) building rapport, (e) conducting quantitative interviews, (f) using arts-based interviewing techniques and methods, (g) conducting cognitive interviews, (h) engaging with children through play, (i) talking about difficult topics and managing distress, (j) referral procedures, (k) developing research questions, (l) using tablets to conduct research, (m) ethical protocols, (n) data protection, (o) COVID-19 protocols, (p) self-care, and (q) aims of the research and content of research tools. Prior to the training, few of the fieldworkers had experience in conducting violence research or research with young children. However, the experiential nature of the training along with the cohesive and open dynamic of the group resulted in a substantial strengthening in research skills of the fieldworkers to collect data on violence and interview children; the training also created an environment where norms and beliefs tied to violence (such as those around gender) were able to be discussed openly and allowed space for shifts in values and perceptions on these topics. The study employed a registered, qualified, full-time social worker, from the same municipal area, who handled all participant referrals and mandatory reporting that arose during data collection.

### Participant Recruitment

Participants comprised three groups which were purposively sampled: young adults, children, and former caregivers. Young adults in the villages were eligible if they were aged 22–30 and had a child aged 4 or older. Children of the young adults were recruited if they were aged 4–7. The young adults’ former primary caregivers (from adolescence)—the sampling strategy to be used in the main longitudinal cohort study—were eligible if they lived locally and were over 35 years old. Former caregivers were ineligible if the young adult indicated that they would struggle cognitively to participate (e.g., they had recently suffered a stroke). As these groups comprised a multi-generational study sample, young adults were the first in the family to be recruited, thereby testing the procedures to be used in the main study.

Young people who met the age criteria were purposively sampled on the street and by going door-to-door. If they expressed interest, participants were informed about the purpose of the study during a face-to-face session in a private place (often the participant’s home) and were provided with and walked through information sheets where the purpose and procedures of the study were further explained. Young adults provided written informed consent for their participation and were then interviewed. Following the interview, if additional children were needed for the child sample, the young adults were asked if their child could participate in an interview. Parents were informed of the circumstances under which mandatory referrals would be made by the fieldworkers prior to being asked to provide consent for their child’s participation and their own participation. This sequence of only recruiting children to the study after their parent had completed the young adult questionnaire sought to ensure the parent understood the scope of the study and nature of the interview. Children for whom parental consent had been obtained were approached, informed about the study aims and procedures, and invited to provide informed assent. If the young adult had an eligible former caregiver, the young adult was invited to contact their caregiver after completing the young adult questionnaire and to seek consent to provide the fieldworkers with their contact information. The fieldworkers then contacted the caregivers to obtain written informed consent.

As a reimbursement for their time, adult participants (young adults; caregivers) received a 50.00 ZAR (2.57 GBP) grocery voucher for each interview in which they participated, and children received a gift pack (valued at 80.00 ZAR (4.10 GBP)) in their first interview (quantitative) which consisted of an activity book, a washcloth, and a soap and were later provided with a small additional gift (stickers; a pen) if they participated in multiple interviews, such as subsequent qualitative interviews. Child participants also received refreshments in the form of a juice box and some biscuits.

### Data Collection

All data were collected on android tablets using the Open Data Kit software, which is an open-source software for collecting, managing, and using data in resource-constrained settings. All participants received a unique identifier number at the point of enrolment into the study, and research data contained no personal identifying information. Data were encrypted and submitted to the KoboToolBox Server—an open-source tool for mobile data collection—where they were stored on an encrypted container and pulled onto a safe server at the University of Edinburgh. All study materials were translated from English into XiTsonga. Translations were then checked and back-translated and discussed among the fieldworker staff until a consensus could be reached that the translations were accurate and retained the meaning of the English items. This consensus-based translation process was highly iterative and required multiple rounds of review by the fieldwork team before there was agreement that the items were translated in a manner that was clear, understandable (appropriately colloquial), and culturally appropriate (Epstein et al., 2015; World Health Organization, n.d.).

Three categories of data were collected from recruited participants in this pilot study: cognitive interview data, quantitative questionnaire data, and qualitative in-depth interview data. Cognitive interviews were conducted to assess the appropriateness of study measures and tools for all sample groups and to determine young children’s (ages 4–7) comprehension and ability to engage with research questions about violence (Centers for Disease Control & Prevention, 2014). In the cognitive interviews, fieldworkers asked participants a question from the quantitative questionnaires and assessed their comprehension of the question and thought process employed in answering the question via follow-up probes (Bell, 2007; Woolley et al., 2004). The aim of the probing questions used was to identify problematic items/measures and determine the suitability of measures for a diverse sample of participants (Collins, 2015; Gray et al., 2014; Willis, 2004; Willis & Miller, 2011). Given the goal of assessing comprehension and cognitive processes, young adult participants outside the age requirements for the rest of the study (aged 22–30 years) could be recruited to participate in the cognitive interviews. For testing measures not related to parenting, young adults without children could also be recruited to the cognitive interviews. The measures tested in cognitive interviews are summarised in Table 1, and an example of a child and adult cognitive interview guide employed in the pilot study is available as Supplemental Table A. Most of the included measures were selected because they were validated and had been previously used in South African settings, though some were not validated for the South African context.

**Table 1:** Measures tested in cognitive interviews

Child measures	Adaptations made for context/age group
Bullying^1^	Language simplified for younger children
Domestic violence exposure^2^	Language simplified for younger children
Parenting (CECPAQ)^3^	Language simplified for younger children
Physical and emotional abuse; sibling abuse and neglect (ICAST-C)^4^	Language simplified for younger children
Community violence^5^	Language simplified for younger children and shortened to include fewer items
Depression (child depression inventory)^6^	Language simplified for younger children
Physical health^7^	Phrasing revised for items asking about hospital admission (overnight hospital stays)
Suicide ideation^8^	Language simplified for younger children and shortened to include fewer items
Resilience^9^	Language simplified for younger children
Trauma^10^	Language simplified for younger children
Adult measures	
(Men) non-partner sexual violence victimization^11^	Inclusion of context-specific form of non-partner sexual violence
(Men) partner and sexual violence perpetration^12^	Revised measure assessing the frequency of partner violence
(Women) partner violence victimization^12^	Revised measure assessing the frequency of partner violence
Resilience^13^	Phrasing revise for items asking about feelings of sadness to match context-specific vocabulary
Childhood emotional abuse, physical abuse, and neglect (ICAST-R)^14^	Inclusion of context-specific examples of physical abuse
Parenting (CECPAQ)^3^	Revised the examples of toys to list common context-specific toys
Parental discipline (ICAST-P)^15^	Inclusion of context-specific examples of physical abuse

^1^(Ruchkin et al., 2004); ^2^(Runyan et al., 2009); ^3^CECPAQ: Comprehensive Early Childhood Parenting Questionnaire (Verhoeven et al., 2017); ^4^ICAST-C: ISPCAN Child Abuse Screening Tool—Child Version (Zolotor et al., 2009); ^5^(Richters & Martinez, 1990); ^6^(Kovacs, 1992); ^7^(WHO, 2010); ^8^(Sheehan et al., 2010); ^9^(Ungar & Liebenberg, 2011; van Rensburg et al., 2019); ^10^(Briere et al., 2001); ^11^(Fulu et al., 2017); ^12^(Fulu et al., 2013; Garcia-Moreno et al., 2005); ^13^(Smith et al., 2008); ^14^ICAST-R: ISPCAN Child Abuse Screening Tool—Retrospective Young Adult Version (Dunne et al., 2009); ^15^ICAST-P: ISPCAN Child Abuse Screening Tool—Parent Version (Runyan et al., 2009)

**Fig. 1:**
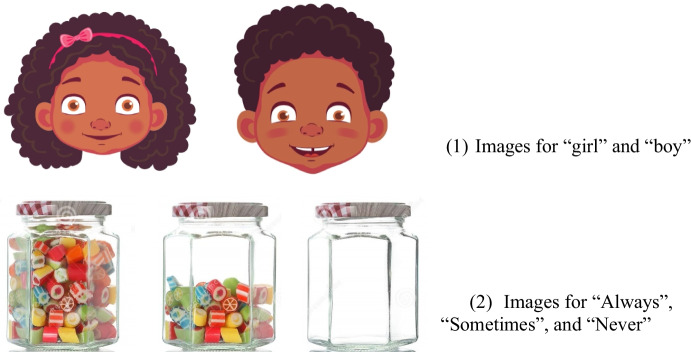
Example images used in children’s questionnaire

Quantitative questionnaires were administered to establish their length and burden on participants, as well as determine if the included measures were appropriate and acceptable. The adult questionnaires (young adult; caregiver) were fieldworker-led with two sections (partner and sexual violence perpetration [men]; partner violence victimisation [women]; non-partner sexual violence victimisation [men; women]) requiring participant self-completion using Audio Computer Assisted Interviewing (ACASI). In these ACASI sections, the participants listened to pre-recorded questions through headphones and responded by selecting their answers on the tablet. The child questionnaire was fieldworker-led and included arts-based activities and tactile and visual props to aid younger children in the response process. The arts-based activities included a facial expressions drawing game (‘feeling faces’) to help children verbalise, identify, recognise, and check for understanding of basic feelings (happy, sad, scared, and angry) and a house plan drawing with playdoh figures to determine children’s living conditions and identify safe and unsafe people and spaces in their home (Fouche & Joubert, 2003). Visual props included a picture-based questionnaire (examples in Fig. 1) which was deployed alongside the tactile props of plastic containers filled, half-filled, or without beans to represent response options (i.e., always, sometimes, and never). Fieldworkers completed fieldnotes at the end of the child interviews to indicate how the child behaved during the interview and how well the child seemed to understand and engage with the interview questions and tasks.

Qualitative in-depth interviews with the children were conducted to assess the suitability of the interview guide and whether young children would be able to understand and meaningfully engage in the interviews. For adult participants, qualitative in-depth interviews were conducted to evaluate the interview guides and determine if adults would participate meaningfully with arts-based interview tasks when discussing topics of violence and families. For both children and adults, we assessed whether participants felt distress or discomfort when discussing violence in an in-depth interview via questions about their feelings when asked about these topics, observational notes recorded by the fieldworkers about their engagement, and displayed verbal and non-verbal cues (shifts in volume of voice, talkativeness, depth of answers, etc.), as well as insight from the study social worker regarding the distress the participants’ reported or the social worker observed at any referral follow-up appointments. Adults participated in two qualitative interview sessions with the fieldworker, which eased the time burden required for adults in a single day and allowed interviewers to reflect between the interviews and to probe in the second interview based on the responses given in the first interview; children participated in one in-depth interview. Fieldworkers employed arts-based methods in the interviews to explore complex and sensitive questions about relationships and violence. These included a squiggle drawing to warm up to the arts-based methods as well as build rapport, the ‘feeling faces’ game (children interviews only), the Road of Life timeline (adult interviews only), and kinetic family drawings used to explore relationships and the feelings between the participant and members of the family. Children created one kinetic family drawing for their current home, and adults created two kinetic family drawings (one for the family of origin and one for their current family). Adult interviews broadly covered parenting, mental health, and coping during childhood in the first interview session and focused more deeply on violence, coping, and HIV in the second interview session. Child interviews explored violence, family relationships, and coping.

Following completion of data collection from participants, three focus group discussions were conducted with fieldworkers to assess implementation and reflect on interviewer concerns and lessons learned. Discussions were audio recorded and transcribed. The investigative team meets weekly to discuss implementation and reflect on data collectively as it was gathered and submitted.

### Data Analysis

Cognitive interviews were transcribed and translated from XiTsonga to English. Transcripts were reviewed by the study team and qualitatively coded to note which items or probing questions participants struggled to understand or respond to in an in-depth manner and which sequencing of items produced the most nuanced and in-depth answers. Quantitative data from the questionnaires were analysed to generate descriptive (frequency, percentage) statistics for all measures using Stata 17 (StataCorp, 2021). Qualitative interviews were transcribed and reviewed by the study team to assess their feasibility with participants and determine interviewer capacity to utilise these methods with participants. Focus group discussions were transcribed and analysed using a rapid analytic approach (Hamilton & Finley, 2019; Vindrola-Padros & Johnson, 2020) for themes around the acceptability, feasibility, appropriateness, and consenting issues of the interview methods and materials and the ability of the methods and materials to facilitate in-depth discussions with the participant around violence, parenting, HIV, and other sensitive topics.

### Ethical Approval

Ethical approval for the pilot study was granted by the University of Edinburgh School of Social and Political Sciences, University of the Witwatersrand Human Research Ethics Committee, North-West University Health Research Ethics Committee, and the Provincial Department of Health Mpumalanga.

### COVID-19 Safety Protocols

As this pilot study took place in the midst of the COVID-19 pandemic, study protocols were employed to ensure the safety of fieldworkers and study staff and all study participants. Interviews took place in well-ventilated areas—typically outside—and participants, and the study team wore masks at all times. All staff and participants adhered to physical distancing per government guidelines. Interview tablets and any other materials that were touched by the study team and participants were sanitised and disinfected after each use. Lateral flow tests were provided to all study staff for regular self-testing, and the whole study team was fully vaccinated during this pilot study. Project policy included a self-isolation protocol if the staff member experienced any cold or COVID-19 symptoms or if any of their family members developed COVID-19 symptoms.

## Results

The results of the pilot study includes data from the cognitive interviews, quantitative questionnaires, qualitative in-depth interviews, and focus group discussions. The findings are presented here in the order in which fieldwork tasks were conducted: cognitive interviewing informed and was followed by quantitative questionnaires which then informed and were followed by in-depth qualitative interviews and focus group discussions. Though new participants were recruited for each of the three pilot study stages, there was some participant overlap between the interview groups. However, given the distinct eligibility requirements for the young adult cognitive interviews and the goals of the pilot study which did not require retaining participants across all three study stages, not all of the participants from the cognitive interviews were invited to the quantitative stage, and not all of the participants from the quantitative questionnaires were invited to the qualitative stage. Twenty percent (*n* = 11) of the participants who took part in the cognitive interviews went on to be recruited and participate in the quantitative questionnaires, and 51% (*n* = 24) of the participants from the questionnaires subsequently were recruited and took part in the qualitative in-depth interviews. Most of the eligible individuals in the community who were approached and invited to the study agreed to participate. Young adults who declined explained that they did so because their time was limited due to a fluid work schedule (‘hustling’, where they would need to be available to start working when an opportunity presented itself). Some children who agreed to participate were not interviewed due to their availability around their school and exam schedules. None of the invited individuals who declined indicated discomfort or disinterest in the study topic. Across the four stages of the pilot study, data were collected on the involvement of the study social worker and their involvement in responding to distress and mandatory reporting procedures required to ethically collect data. Findings related to the social worker’s involvement are also presented, as the social worker’s participation informed the findings of the pilot study regarding the feasibility of collecting violence data from young children and across family generations in an ethical manner that minimised harm and distress.

### Cognitive Interviews

Cognitive interviews were conducted with each of the three sample groups: children, young adults, and caregivers. Twenty-four children, 23 young adults, and nine caregivers participated in cognitive interviews. Demographic information for cognitive interview participants is presented in Table 2. Two or three cognitive interview measures (Table 1) were assessed per participant for participant comprehension and measure acceptability.

**Table 2:** Demographic characteristics of the child (*n* = 24), young adult (*n* = 23), and caregiver (*n* = 9) participants for the cognitive interviews

Children		Young adults		Caregivers	
Gender	*n* (%)	Gender	*n* (%)	Gender	*n* (%)
Boy	10 (42)	Man	5 (22)	Man	2 (22)
Girl	14 (58)	Woman	18 (78)	Woman	7 (78)
Age		Age		Age	
4	12 (50)	21–24	14 (61)	40–49	6 (67)
5	5 (21)	25–27	6 (26)	50–59	3 (33)
6	5 (21)	28–30	2 (9)		
7	2 (8)	Unknown	1 (4)		
Enrolled in:		Highest education		Highest education	
Crèche or school	18 (75)	Some primary	1 (4)	Some primary	2 (22)
Neither	6 (25)	Some secondary	9 (39)	Some secondary	3 (33)
		Grade 12	5 (22)	Grade 12	4 (45)
		Matric	2 (9)	Matric	0 (0)
		Any tertiary	5 (22)	Any tertiary	0 (0)
		Unknown	1 (4)		

#### Children

For the children’s cognitive interviews, some children struggled to understand certain terms. For example, when asked about ‘admission’ to hospital, the children explained their understanding of this question to mean any routine hospital visit, such as vaccination appointments. Additionally, some items in the bullying measure caused confusion such as the item that asked if anyone ever ‘Made me uncomfortable standing too close or touching me’. In these instances of misunderstanding or potential ambiguity, the phrasing of the items was modified. For instance, the hospital item was rephrased to ask if they ever had to ‘stay in hospital for at least one night’ and the bullying item became ‘Tried to make me scared by standing too close or touching me’; both of which were found to clarify the meaning for the participants. Children were comfortable with all the tested items in the cognitive interviews and particularly enjoyed the arts-based activities (drawing tasks; facial expressions game) that accompanied the measures. Overall, the measures were found to be acceptable among all the children and comprehension of the measures was good.

The children’s individual cognitive development and verbal ability—more than their specific age—influenced their ability to participate in the cognitive interviews of the items. For example, while several of the youngest children (aged 4 and 5) struggled to express themselves in their cognitive interviews, other children this same age who were more developmentally advanced were able to express their answers and understanding of the items with more complexity and nuance. Conversely, while most of the 7-year-olds found the cognitive interviews easier to engage in than the youngest children, some of these older children were developmentally similar to the 4- and 5-year-olds and faced similar limitations in their explanations. Overall, we found that nearly all the children interviewed were able to understand the items, repeat them back to the fieldworkers, and answer them with confidence, even providing specific detail in their answers to indicate their comprehension. However, many of the children were not developmentally advanced enough to participate in the in-depth cognitive probing in which they explained their thinking and reasoning around their answers.

#### Adults

For the adults, most of the items in the tested measures were well understood, though some specific vocabulary proved confusing. Some participants with children were not able to relate the items that asked about games and toys they played with their children (parenting measure: CECPAQ) because the examples provided—colourful building blocks, rattle books, Lego, etc.—were uncommon in their community. Based on this finding, the examples listed in these items were changed to reflect toys common to the study setting, including tins, cars, and soft toys. Metaphorical expressions, such as ‘I’m on my child’s back’ (from the parenting measure), also caused confusion and were adjusted to read ‘I’m more critical than usual’ to clarify. Similarly, the resilience measure was adjusted after it was found that some participants understood ‘set-back’ and ‘bounce back’ to have the same meaning. This was corrected by adjusting the translations from English to be more descriptive and explain setbacks as ‘difficulties or challenges that are hard to overcome’ and bouncing back as ‘returning to feeling like myself after hard times’. Participant feedback indicated that adults found most of the items acceptable. Some adults were initially uncomfortable speaking about the age of their first sexual encounter or their HIV status, but they were willing to answer the questions and continue the interview. For the violence items, men and women were comfortable being interviewed by fieldworkers who were both men and women about their personal violence victimisation and/or perpetration experiences. Overall, the tested measures were acceptable and well understood among the adult cognitive interview participants, and only minor vocabulary changes were required for the quantitative questionnaire items.

### Quantitative Interviews

Quantitative interviews were conducted with 21 young children, 29 young adults, and 11 caregivers. This quantitative interview sample included four children, six young adults, and one caregiver who also participated in the cognitive interviews. Demographic characteristics of the quantitative participants are summarised in Table 3.

**Table 3:** Demographic characteristics of the child (*n* = 21), young adult (*n* = 29), and caregiver (*n* = 11) participants for the quantitative interviews

Children		Young adults		Caregivers	
Gender	*n* (%)	Gender	*n* (%)	Gender	*n* (%)
Boy	11 (52)	Man	8 (28)	Man	2 (18)
Girl	10 (48)	Woman	21 (72)	Woman	9 (82)
Age		Age		Age	
4	2 (10)	22–24	12 (41)	40–49	4 (36)
5	7 (33)	25–27	12 (41)	50–59	6 (55)
6	7 (33)	28–30	5 (17)	60–69	1 (9)
7	5 (24)				
Enrolled in:		Highest education		Highest education	
Crèche	7 (33)	None	0 (0)	None	6 (55)
School	9 (43)	Some primary	3 (10)	Some primary	2 (18)
Neither	5 (24)	Some secondary	13 (45)	Some secondary	2 (18)
		Matric	8 (28)	Matric	1 (9)
		Any tertiary	5 (17)	Any tertiary	0 (0)
		Citizenship		Citizenship	
		South Africa	26 (90)	South Africa	8 (73)
		Mozambique	3 (10)	Mozambique	3 (27)

#### Intergenerational Consent Procedures

All of the young adult participants who lived with their eligible child provided consent for their child to participate following the young adult quantitative interview. Some parents were concerned that their child would find the questionnaire too long and confusing. Once they were reassured that the children were interviewed using a shorter and child-friendly questionnaire covering the research topics, the originally hesitant parents provided consent. This consent procedure worked well; information about the circumstances under which mandatory referrals would be made was embedded in the invitation to provide parental consent, and no parents expressed hesitancy or denied their consent when informed of this requirement. All parents who were invited to provide parental consent for their children did so, resulting in no refusals from young adults for their children to participate. All young adults with eligible former caregivers living in the study area provided consent for the fieldworkers to contact them, and all contacted caregivers consented to participating.

#### Children

The child questionnaire took approximately 1 to 2 h to administer, which included the arts-based activities. The young children participating were able to complete the interview with minimal loss of focus. Children’s focus and active engagement during the interview were likely helped by the design of the questionnaire, which included play-based and arts-based activities and questions as well as tactile and visual aids incorporated into the questionnaire items to assist engagement, comprehension, and focus.

For children, the key findings from the quantitative interviews were that the visual prompts, tactile aids, and arts-based activities were well-received. Most children were able to enthusiastically participate for the full length of the interview. When children struggled to remain focused, the fieldworkers were trained to take a break and provide age-appropriate accommodations. These included frequent breaks from the questionnaire to draw, to play games or run around outdoors, to talk or sing, or to have a snack. Children often returned from these play breaks more focused and ready to continue with the interview. On other occasions, children wanted to continue answering the questions while being allowed to draw, build with playdoh, walk around with the fieldworker, or engage in a different manner that involved movement and allowed the child to do more than sit quietly and answer questions. Most of the children were able to comprehend the questions in the interview and speak knowledgably about their life, including around experiences of violence. The children who were less developmentally advanced sometimes struggled most with focus and had some issues with understanding the more complex questions (e.g., the question about their community ‘People make fun of your situation’), but play-based accommodations from the fieldworkers and additional explanation of the items allowed these children to fully participate and complete the interview.

#### Adults

The first version of the adult questionnaires (young adult; caregiver) administered in the pilot study took over 4 h to complete. The questionnaires were then abridged, and response times were shortened by approximately an hour. Interviews with the women were typically completed in 2 to 3 h, while interviews with the men lasted 3 to 4 h, despite the men having slightly fewer items asked in their interview. Our fieldworkers noted that the men were often enthusiastically engaged in the interviews and wanted to have follow-up discussions regarding the topics in the questionnaire (e.g., coercion in the context of a romantic partnership). Additionally, some of the men were relatively uninvolved fathers—rarely playing with and/or disciplining their child; not attending health clinic visits with their child and/or not being knowledgeable about the health history of their child—which meant they struggled to answer the questions related to parenting and their relationship with their child. Fieldworkers noted that this mix of additional engagement for some men and lack of parental involvement for some men resulted in longer average interview times compared to the women’s interviews.

The quantitative interviews with the adult questionnaires were well-received and presented few issues, particularly once the interviews were shortened via abridged questionnaires. Very few items in either the young adult or caregiver questionnaire prompted participants to refuse to answer, and no clear pattern or trends emerged around which items participants refused. Fieldworkers collected participants’ perspectives on the questionnaire items via exit questions administered at the end of the interview. These results are summarised in Table 4. The majority of adult participants did not find the questionnaire items cognitively difficult, were not upset by the items asked in the questionnaire, believe the items asked about are important research issues, and would be willing to participate in the interview again now that they know what would be asked. Our fieldworker team—composed of women and one man—stated that they were each able to interview all participants, regardless of gender, and have frank discussions on sensitive topics without fieldworker discomfort or reported or visible discomfort by the participants.

**Table 4:** Responses to exit questions answered by young adult (*n* = 29) and caregiver (*n* = 11) participants

Young adults		Caregivers	
	*n* (%)		*n* (%)
How difficult were the items in the questionnaire?		How difficult were the items in the questionnaire?	
Not at all difficult	22 (76)	Not at all difficult	6 (55)
A bit difficult	5 (17)	A bit difficult	4 (36)
Very difficult	2 (7)	Very difficult	1 (9)
How upset were you by the items asked?		How upset were you by the items asked?	
Not at all	26 (90)	Not at all	10 (91)
A little bit	2 (7)	A little bit	0 (0)
Very upset	1 (3)	Very upset	1 (9)
Do you think the research issues included in the questionnaire are important?		Do you think the research issues included in the questionnaire are important?	
Not at all important	0 (0)	Not at all important	0 (0)
A little bit important	1 (3)	A little bit important	0 (0)
Very important	28 (97)	Very important	11 (100)
Would you be willing to complete this questionnaire again?		Would you be willing to complete this questionnaire again?	
Yes	28 (97)	Yes	10 (91)
No	1 (3)	No	1 (9)

### Qualitative Interviews and Focus Group Discussions

Qualitative interviews were conducted with 18 young children, 22 young adult, and seven caregiver participants. The children’s interviews lasted approximately 45–60 min, including the drawing task. Adults participated in two qualitative interviews with first session typically lasting 1 h and the second session 45 min. Demographic characteristics of these participants are summarised in Table 5.

**Table 5:** Demographic characteristics of the child (*n* = 18), young adult (*n* = 22), and caregiver (*n* = 7) participants for the qualitative interviews

Children		Young adults		Caregivers	
Gender	*n* (%)	Gender	*n* (%)	Gender	*n* (%)
Boy	9 (50)	Man	6 (27)	Man	1 (14)
Girl	9 (50)	Woman	16 (73)	Woman	6 (86)
Age		Age		Age	
5	6 (33)	22–24	6 (27)	40–49	3 (42)
6	4 (22)	25–27	9 (41)	50–59	2 (29)
7	7 (39)	28–30	7 (32)	60–69	2 (29)
8	1 (6)				

#### Children

Young children were able to participate and answer most of the qualitative interview questions. A few children struggled to understand questions exploring resilience (fieldworkers would start by asking ‘What makes you strong?’), as they understood questions to be asking about physical strength. The fieldwork team revised the phrasing of this question to clarify that the question meant emotionally strong (asked as ‘strong in your heart and mind’). All of the young children interviewed enthusiastically engaged in the interviews and enjoyed having conversations with adults who were attentively listening to them. As in the quantitative questionnaires, some of the children struggled to maintain focus for the whole interview, so fieldworkers provided play breaks and flexible accommodations to help the children regain focus. The children did not demonstrate emotional distress or dysregulation in the interviews, even when disclosing difficult experiences. Instead, the fieldworkers observed (and in some cases, the children reported) that they enjoyed the chance to talk about these difficult topics with an adult who listened to and believed them. The children who were less verbally advanced (often the youngest of the interviewed sample but not always) enjoyed participating but tended to provide less rich narratives and required more play breaks during the interview, compared to children more developmentally advanced and with more language skills.

#### Adults

The adult participants enjoyed the interview and the arts-based elements incorporated. Some adults struggled to understand probing questions about coping and resilience (The two difficult questions were ‘How do families cope during difficult times?’ and ‘What makes families strong?’). Based on fieldworker observations and participant reports, some of the adults were distressed during the interviews. However, probing by the fieldworker and follow-up visits by the social worker made it clear that they were not distressed about being asked the interview questions; rather, their distress stemmed from the difficulties they were discussing in the interview, such as hardships or violence at home or in their relationships. Irrespective of their sample group (young adults; caregivers), adults provided rich narratives and valuable data regarding their experiences with violence, mental health struggles, parenting, and HIV.

### Social Worker

A full-time social worker was employed during the pilot study to handle participant referrals and mandated reports. Automatic referral flags were produced during the quantitative interviews if a participant disclosed behaviours, thoughts, or feelings of concern. Fieldworkers could also create a referral if they observed anything of concern during any of the interviews such as participant distress. Participants were also asked if they would like to speak to a social worker at the end of each interview, and those who responded affirmatively were referred. The social worker was responsible for assessing, containing, and following up with all participants who were referred for any reason during the pilot study. The social worker provided needed counselling, connected participants to services available in the setting, as needed, and completed any mandatory referrals to statutory social services with follow-up support.

Table 6 summarises the automatic referral flags generated based on quantitative interview responses. No emergency referrals were made for a child who was in immediate danger. Additionally, any participants who expressed distress during their interview were able to be contained by the fieldworker speaking to them; though some of these distressed participants were referred to the social worker for follow-up, none of these distress referrals were an emergency. All referrals were investigated by the social worker, who dealt with statutory referrals first before providing support for non-mandatory referrals based on urgency. The management of cases included referrals to the Department of Social Development (DSD), on-site counselling, referrals for longer-term counselling and assistance with food parcels, and referrals to the Department of Home Affairs for birth registration and identity documentation. For more information on the management of referred cases, see Supplemental Table B.

**Table 6:** Count of referral codes generated in quantitative interviews with child (*n* = 21), young adult (*n* = 29), and caregiver (*n* = 11) participants

Referral code	Children	Young adults	Caregivers
	*n*	*n*	*n*
Adult sexual violence	—	7	1
Anxiety symptoms	10	—	—
Bilharzia	0	1	0
Bullying (by peers)	19	—	—
Bullying (by siblings)	15	—	—
Child sexual abuse	1	0	0
Depression symptoms	7	—	—
Emotional abuse	13	—	—
Food insecurity	6	—	—
Harsh parenting/physical discipline	—	22	2
Intimate partner violence/domestic violence	9	10	0
Physical abuse	15	—	—
Psychosis	—	3	2
Self-referral	—	16	9
Suicidality	0	6	2
Trauma symptoms	11	—	—
Tuberculosis symptoms	—	7	3

— indicates ‘not applicable’ to this sample group

Along with handling participant referrals, the social worker was a helpful resource to the fieldwork staff. Fieldworkers reported feeling more comfortable asking sensitive questions and less at risk for secondary trauma because they knew participant needs would be handled by the study social worker. The training and management of the study team followed a trauma-informed approach in which self-care was a prominent focus, and the risk for secondary trauma was discussed among the team members (Purtle, 2020; Sexual Violence Research Initiative, 2015). The social worker provided assistance in these self-care practices, which also included team debriefs and reflective sessions around the difficult disclosures they were handling and regular self-care practices at team meetings. Together, the involvement of the social worker and trauma-informed management seemed to be effective, as our fieldworkers reported that they felt well-prepared to cope with difficult participant disclosures and secondary trauma due to the team resources available and employed self-care practices.

## Discussion

This paper described the key findings of a pilot study sampling three generations of family members in rural villages of Mpumalanga province, South Africa. The primary aim of the pilot study were to assess the length, participant burden, and appropriateness the measures and to determine if young children (aged 4–7) could meaningfully participate in quantitative and qualitative research about violence. The pilot study also sought to determine the feasibility of recruiting multiple generations of participants, particularly children, into a study investigating intergenerational violence and whether such a study would be ethically sound. Specifically, the pilot study is aimed at understanding if multiple generations could safely participate in interviews on sensitive topics, if the measures used were suitable, if young children could feasibility participate in research on sensitive topics, and if the study procedures would appropriately protect participants from distress or harm.

The length of the interviews proved feasible for both children and adults. Children were able to stay focused and engaged throughout their interviews, and fieldworkers took breaks to play with the children if their focus started to drift. Adult participants were willing to engage in the lengthy quantitative (2–4 h) and qualitative (2 h) interviews. However, in an effort to reduce the burden on participants, additional items will be cut from the quantitative questionnaire to decrease the interview length by 30–60 min in the subsequent main study. The cognitive interviews highlighted some need for rewording or retranslation in order to clarify the meaning of the question or to include culturally appropriate reference points (e.g., which toy examples are listed when asking about parenting and play).

Study findings demonstrated that young children are able to participate in rigorous quantitative and qualitative research asking about violence when visual aids, tactile props, and arts-based methods are woven into the research methods and interview structure. Young children were found to be valid and reliable reporters of their experiences surrounding violence and other sensitive topics, such as HIV. For example, the patterns of household violence or family strain reported by the young adults were most often also reported by young children, demonstrating that children were aware of the violence and stressors in the home and were able to accurately describe and report these in an interview. The youngest of the children (4- and 5-year-olds) were able to engage in the research, though the qualitative data most of these youngest children provided tended to be less rich, as they were typically less developmentally advanced than those aged 6 and 7. However, some of the youngest children (aged 4) surpassed the verbal and cognitive development of some of the oldest children (aged 7) and provided rich and detailed qualitative data. While children were able to understand and answer the items in the cognitive interviews, many were not developmentally advanced enough to engage in the abstract thinking required to articulate the reasoning that led to their answers. This is developmentally appropriate and to be expected from children aged 4–7 (Bell, 2007; Cameron, 2005; Otsuka & Jay, 2017; van Oers & Poland, 2012; Woolley et al., 2004).

The ability for the children to remain focused and engaged during the interviews also depended on their development, as those in the earlier developmental stages often struggled more to maintain focus during the interviews, though this was handled via play-based accommodations. Ensuring that children were not expected to sit quietly and answer questions for an extended period but were offered movement and accommodations via art and play constituted a key study feature aiming to make the young child interviews child-centred (Koller & San Juan, 2015; O’Reilly & Dogra, 2017). Some fieldworkers were less prepared to meet the less developed children where they were and initially struggled to find ways to engage the children who required significant play, flexibility, and accommodations. These findings demonstrated that fieldworker skills and training were an essential element in conducting this research with such young children with a diversity of developmental stages. We believe it is likely that we would have had fieldworkers who struggled less and were better prepared to engage with children at their level had we hired fieldworkers with more expertise and knowledge in early child development and had we provided additional in-depth training and education around early childhood. This fits with previous findings that the quality of interview data from child participants depends on the skill of the interviewer modifying their approach to fit the needs of the specific child and their engagement (Danby et al., 2011). As knowledge of cognitive and other developmental features of childhood stages is essential to selecting and utilising appropriate interview techniques when working with a young child, fieldwork staff skilled in understanding the stages of development in early childhood, knowing the normal abilities of each stage, and being able to provide appropriate play and accommodations within each stage would be able to conduct this research and obtain quality data with all the children in our sample (Caldairou-Bessette et al., 2020; Cameron, 2005). We found it essential to provide mentoring that focused on value clarification to better equip our fieldworkers to interview young children. Given the common context-specific values regarding children (e.g., that they should be seen but not listened to; that they should not speak back to adults or express their opinion), we sought to be intentional in this mentorship to challenge these beliefs and encourage our fieldworkers to consider the benefits of listening to and believing young children. The value clarification increased the interview skills among our fieldworkers, and those who were most open to the process became the most skilled at engaging which children at their level and introducing elements of playfulness into their interviews. Given our staff’s level of preparation and our ability to train and increase their skills in this area, we made the decision to adjust the child age of eligibility to 6 years old and above for the main study. However, we are confident that similar arts-based, mixed-methods research about violence and/or other sensitive topics could be done with children aged 4 and 5 if the fieldwork staff were trained and supported around early childhood development and accommodations.

Children and adults enjoyed the arts-based elements in the quantitative (children only) and qualitative (children and adults) interviews. Prior to completion of the pilot study, some of the involved ethical committees were sceptical that children would be able to produce meaningful arts-based data and that adults would be willing to engage in the arts-based tasks. Our findings demonstrated that our participants enjoyed the tasks and enthusiastically participated regardless of their age—from the youngest child to the oldest caregiver—and that each group was able to produce high-quality data regardless of the artistic ability of the participant. We found the arts-based methods to be extremely valuable in exploring sensitive topics for both children and adults, despite initial reservations from the ethics committees (Betts, 2006; Bunn et al., 2020; Carter & Ford, 2013; D’Amico et al., 2016; Stuckey & Nobel, 2010).

This pilot study also demonstrated that the study procedures and policies were successfully able to recruit participants for a three-generation study using comprehensive consent protocols which included informing participants of mandatory reporting which required following certain disclosures. When considering parental consent for their child’s participation, young adult participants sought reassurance that the child would not be asked the same complex adult items they answered and were willing to provide parental consent once fieldworkers informed them that the questionnaires were shorter and age-appropriate for young children. Young adults were also comfortable with the required referrals to the social worker and approached these favourably, often viewing them as an opportunity to seek help from a social worker.

Social worker referrals were not required due to the distress from study participation for any of the sample groups. Referrals frequently reflected the social issues experienced by the communities in the study, such as the common self-referrals for assistance with poverty or identity documentation. As a result, participant engagement in the study provided access to services to address unmet needs and access usually hard-to-reach social services.

No adverse effects were reported by the participants, observed by the fieldworkers, or noted by the study social worker regarding the involvement of multiple family generations in a study examining sensitive and difficult topics. The study procedures for consent, privacy and confidentiality, and safeguarding seemed to adequately protect individuals from potentially harmful effects via participation. Privacy was especially crucial to protect young children during participation, as some parents/primary caregivers tried to stay nearby or listen through walls in order to overhear the child’s interview. This attempt to eavesdrop by parents likely reflects a curiosity about their child’s answers and, for some parents, a desire to control their disclosures. Some parents seemed keen to have their child perform ‘well’ and give the ‘right’ answers in the interview—though fieldworkers emphasised to the child that they were interested in their opinion and that there were not correct and incorrect answers to the questions—and other parents likely wanted to know what was discussed given the mandatory reported requirements communicated to them. This aligns with findings that gatekeepers often seek to block or control children’s participation in research, in this case by allowing the child to participate but seeking to overhear and influence their answers (Shier, 2023). Training on the essential nature of privacy during interviews ensured that the staff sought a private space to conduct the interviews or social worker follow-up visits and that they had the confidence to ask family members to step away, as needed, to allow the child to speak privately to the fieldworker or social worker.

### Key Implication for Future Research

This study demonstrated the feasibility of including young children in violence research using ethically sound and age-appropriate procedures and methods. It also demonstrated that multi-generational research regarding violence can be conducted and can include young children if rigorous consent procedures, referral protocols, and participant protections are employed. It is critical that fieldworkers collecting data on violence or working with young children need to be appropriately trained to collect sensitive data and work with vulnerable populations in a rigorous and trauma-informed manner. Additionally, the management of fieldworkers collecting sensitive data needs to be trauma-informed in order to recognise and mitigate risk for secondary trauma among the study team. In demonstrating that, via carefully designed procedures and tools, young children can participate in sensitive quantitative and qualitative research, including research about their experiences of violence; this work builds on the findings of Devries and colleagues (2015) which explored how to ethically collect violence data from children in a resource-poor setting while ensuring that children were provided with needed and appropriate support. Based on our experience, we believe that a full-time social worker as a member of the study staff is a critical element when conducting violence research among vulnerable populations, including young children and their families. Though rigorous procedures and ethical protections are required as part of the study protocol, future researchers should not be dissuaded from including young children in violence research because of concerns that parental consent/child assent cannot be obtained, children cannot understand or engage with the research, or young children are not able to provide rich, meaningful data on their experiences of violence.

The arts-based methods used in this pilot study were immensely valuable in conducting sensitive violence research and seem to be acceptable and enjoyable for both children and adults. Future research into how arts-based methods can facilitate difficult disclosures or provide rich data for less verbally and cognitively developed children would be valuable.

### Limitations

As these findings come from a pilot study preceding a multi-year main study, the sample sizes for each of the study groups were not very large (*n*_child_ = 18–24; *n*_young adult_ = 22–29; *n*_caregiver_ = 7–11). These findings were also limited by the depth of the qualitative interviewing skills of some of the fieldwork staff who previously had conducted only quantitative research. Despite the extensive initial training and multiple skills-building training sessions throughout the pilot study, fieldworkers were quite bound by the probing questions provided in the interview guides for the cognitive interviews and qualitative in-depth interviews and struggled to be adaptable in these interviews and ask unique probing questions based on participant responses. This limitation has informed some decision-making around staffing for the main study, particularly around who will be appointed to conduct the qualitative in-depth interviews.

It is possible that our findings around appropriateness of the measures were influenced by a social desirability bias felt by the participants. Though we sought to analyse, interpret, and present the findings of the pilot study objectively, interpretative bias in these results is possible, as these findings were collected by a team of researchers and staff associated with the main study which would be informed by these pilot study findings. The number of referrals to the study social worker and management of the referred cases may also reflect the context in which the pilot study took place. Some of the referrals were difficult to address due to the structural limitations experienced by existing services. For example, our social worker struggled to meet the needs of participants needing birth or identity documents—particularly among participants who were cross-border migrants—given the resource constraints of the required services to meet these needs. The pilot sample also came from one region in the Mpumalanga province of South Africa, which may limit the scope to which these feasibility and acceptability findings can be applied. However, given the aims of the pilot study were to investigate the methods and measures to be used in the main study, we believe that the key findings around the feasibility of including young children in violence research and recruiting for a multi-generational violence study are applicable beyond this sampled community and beyond South Africa.

## Conclusions

This study demonstrated the feasibility of including young children in violence research and of collecting multi-generational family data focused on intergeneration violence while ensuring that ethical standards were maintained. It also highlighted the value of doing an extensive, in-depth pilot study, as the rich and nuanced findings from the 4-month pilot study greatly informed the tools and protocols of the subsequent main study. Children and adults were willing to participate in interviews covering sensitive topics and found the measures to be comprehensible and acceptable. Children aged 4 and older are valid and reliable reporters of their experiences of violence, though less developmentally advanced children required more play-based accommodations to remain engaged in interviews than their more verbally and cognitively advanced peers. Arts-based techniques were found to be helpful for and enjoyed by child and adult participants, and children were also helped to focus and express their answers using visual prompts and tactile props during the interviews. In order to respond to participant needs and required referrals that arise during sensitive research, we recommend that any study seeking to interview young children or collect multi-generational data employs a full-time social worker as a member of the study staff. Future research will be benefitted by including young children and their voices in studies seeking to understand violence against children or intergenerational violence.

## Supplementary Information

Below is the link to the electronic supplementary material.

Supplementary file1 (DOCX 18 KB)

Supplementary file2 (DOCX 15 KB)

## Data Availability

The datasets generated during and analysed during the current study are not publicly available because they represent preliminary (pilot) data as part of an on-going research study but are available from the corresponding author on reasonable request.
