# Supplementary material for: Conducting Violence Research Across Multiple Family Generations and with Young Children: Findings from a Mixed-Methods Pilot Study in South Africa
Source: Int J Child Maltreat. 2023 Mar 7:1–27. Online ahead of print. doi: 10.1007/s42448-023-00157-w (PMC9988603; doi:10.1007/s42448-023-00157-w)
Supplement: Supplementary file 1 — Supplementary file1 (DOCX 18 KB) [file 42448_2023_157_MOESM1_ESM.docx]

**Supplemental Table A: Examples of cognitive interview guides used in the pilot study to test the items for attitudes about family violence (child interview) and physical abuse (adult interview)**

| **Number** | | **Question** | **Response options** |
| --- | --- | --- | --- |
| **CHILD COGNITIVE INTERVIEW EXAMPLE**  **Attitudes About Family Violence (AAFV)** (Graham Bermann 1994) adapted | | | |
| When children hear arguments and fighting in the home it can be scary. We are now going to ask you what you think about adults fighting. | | | |
| 3.38 | In most families there is a lot of fighting. | | always (2); sometimes (1); never (0) |
|  | Ask question in the questionnaire (**above**) then probe:  1. What is this question asking?  a. What do you understand “fighting” to mean?  b. Can you repeat the question I asked you in your own words?  2. How did you arrive at the answer that you gave?  a. What did you think about before you gave your answer?  b. How did you go about remembering this?  c. How easy or hard was it to give the answer?  3. What influenced you to choose the answer that you gave? | | |
| 3.39 | It’s the kids’ fault when adults fight. | | always (2); sometimes (1); never (0) |
|  | Ask question in the questionnaire (**above**) then probe:  1. What is this question asking?  a. Can you repeat the question I asked you in your own words?  2. How did you come to/arrive at the answer that you gave?  a. What did you think about before you gave your answer?  b. How sure are you about the answer that you gave?  c. How easy or hard was it to give the answer?  3. What influenced you to choose the answer that you gave? | | |
| 3.40 | When people get hit they always deserve it. | | always (2); sometimes (1); never (0) |
|  | Ask question in the questionnaire (**above**) then probe:  1. What is this question asking?  a. What do you understand “hit” to mean? [how is this the same or different from “fight”]  b. Can you repeat the question I asked you in your own words?  2. How did you come to/arrive at the answer that you gave?  a. What did you think about before you gave your answer?  b. How sure are you about the answer that you gave?  c. How easy or hard was it to give the answer?  3. What influenced you to choose the answer that you gave? | | |
| 3.41 | Kids can't do anything when adults fight. | | always (2); sometimes (1); never (0) |
|  | Ask question in the questionnaire (**above**) then probe:  1. What is this question asking?  a. Can you repeat the question I asked you in your own words?  2. How did you come to/arrive at the answer that you gave?  a. What did you think about before you gave your answer?  b. How easy or hard was it to give the answer?  3. What made you choose the answer that you gave? | | |
| 3.42 | Most kids feel angry or sad when parents (adults) fight. | | always (2); sometimes (1); never (0) |
|  | Ask question in the questionnaire (**above**) then probe:  1. What is this question asking?  a. Can you repeat the question I asked you in your own words?  2. How did you come to/arrive at the answer that you gave?  a. What did you think about before you gave your answer?  b. How did you go about remembering this?  c. How easy or hard was it to give the answer?  3. What influenced you to choose the answer that you gave? | | |
| 3.43 | Fighting is the only way to solve problems. | | always (2); sometimes (1); never (0) |
|  | Ask question in the questionnaire (**above**) then probe:  1. What is this question asking?  a. Can you repeat the question I asked you in your own words?  2. How did you come to/arrive at the answer that you gave?  a. What did you think about before you gave your answer?  b. How sure are you about the answer that you gave?  c. How easy or hard was it to give the answer?  3. What influenced you to choose the answer that you gave? | | |
| 3.44 | Children must tell someone about fighting in the house? | | always (2); sometimes (1); never (0) |
|  | Ask question in the questionnaire (**above**) then probe:  1. What is this question asking?  a. Can you repeat the question I asked you in your own words?  2. How did you come to/arrive at the answer that you gave?  a. What did you think about before you gave your answer?  b. How sure are you about the answer that you gave?  c. How easy or hard was it to give the answer?  3. What influenced you to choose the answer that you gave? | | |

| **Number** | **Question** | | **Response options** |
| --- | --- | --- | --- |
|  | **ADULT COGNITIVE INTERVIEW EXAMPLE**  **Physial Abuse: ICAST-R (Emotional Abuse)** | | |
| 8.1.48 | When you were growing up (before age 18), did an adult discipline you by hitting or smacking you? | yes (1); no (0); can't remember (2) | |
|  | Ask question in the questionnaire (**above**) then probe:  1. What is this question asking?  a. Can you repeat the question I asked you in your own words?  2. How did you come to/arrive at the answer that you gave?  a. What did you think about before you gave your answer?  b. How did you go about remembering this?  C. How easy or hard was it to give the answer?  3. What influenced you to choose the answer that you gave? | | |
|  | *[if the participant answers “no” or* “*can’t remember”]*  You answered“no” or “can’t remember“, I would still like your opinion on whether our questions are understandable. I am going to ask some follow up questions, and we would like to know how you understand the question and whether you think the question is acceptable. You may also consider how you would go about answering the question if it had happened to you. | | |
| 8.1.49 | At what times in your life did this happen to you? [select all that apply] | Before you went to school (1); while you were at primary school (2); when you were at high school (3) | |
|  | Ask question in the questionnaire (**above**) then probe:  2. How did you come to/arrive at the answer that you gave?  a. What did you think about before you gave your answer?  b. How did you go about remembering this?  c. How sure are you about the answer that you gave?  d. How easy or hard was it to give the answer?  3. To what extent did the answer options match what your experience / thoughts were when you heard the question? | | |
| 8.1.50 | How often did this happen to you during your life? | too many times to count (1); between 10 to 50 times (2); less than 10 times (3) | |
|  | Ask question in the questionnaire (**above**) then probe:  1. How did you come to/arrive at the answer that you gave?  a. What did you think about before you gave your answer?  b. How did you go about remembering this?  c. How sure are you about the answer that you gave?  d. How easy or hard was it to give the answer?  2. What influenced you to choose the answer that you gave? | | |
| 8.1.51 | Which people did this to you? | Parent (1), step-parent (2), close relative (3), teacher (4), other (5)___specify | |
|  | Ask question in the questionnaire (**above**) then probe:  1. How did you come to/arrive at the answer that you gave?  a. What did you think about before you gave your answer?  b. How did you go about remembering this?  c. How sure are you about the answer that you gave?  d. How easy or hard was it to give the answer?  2. What influenced you to choose the answer that you gave?  3. To what extent did the answer options match what your experience / thoughts were when you heard the question? | | |
| 8.1.52 | How much did this experience hurt or harm you? | a great deal (1); seriously (2); mildly (3); not at all (4) | |
|  | Ask question in the questionnaire (**above**) then probe:  1. What is this question asking?  a. Can you repeat the question I asked you in your own words?  2. How did you come to/arrive at the answer that you gave?  a. What did you think about before you gave your answer?  b. How did you go about remembering this?  c. How sure are you about the answer that you gave?  d. How easy or hard was it to give the answer?  3. What influenced you to choose the answer that you gave?  4. To what extent did the answer options match what your experience / thoughts were when you heard the question? | | |
| 8.1.53 | When you were growing up (before age 18), did an adult ever hit or punch you very hard? | yes (1); no (0); can't remember (2) | |
|  | Ask question in the questionnaire (**above**) then probe:  1. What is this question asking?  a. Can you repeat the question I asked you in your own words?  2. How did you come to/arrive at the answer that you gave?  a. What did you think about before you gave your answer?  b. How did you go about remembering this?  c. How sure are you about the answer that you gave?  d. How easy or hard was it to give the answer?  3. What influenced you to choose the answer that you gave? | | |
| 8.1.58 | Before you were age 18, did an adult ever kick you very hard? | yes (1); no (0); can't remember (2) | |
|  | Ask question in the questionnaire (**above**) then probe:  1. What is this question asking?  a. Can you repeat the question I asked you in your own words?  2. How did you come to/arrive at the answer that you gave?  a. What did you think about before you gave your answer?  b. How did you go about remembering this?  c. How sure are you about the answer that you gave?  d. How easy or hard was it to give the answer?  3. What influenced you to choose the answer that you gave? | | |
| 8.1.63 | Before you were age 18, did anyone ever beat you very hard with an object like a stick, cane, whip, sjambok or belt? | yes (1); no (0); can't remember (2) | |
|  | Ask question in the questionnaire (**above**) then probe:  1. What is this question asking?  a. Can you repeat the question I asked you in your own words?  2. How did you come to/arrive at the answer that you gave?  a. What did you think about before you gave your answer?  b. How did you go about remembering this?  c. How sure are you about the answer that you gave?  d. How easy or hard was it to give the answer?  3. What influenced you to choose the answer that you gave? | | |
| 8.1.68 | Before you were 18, did anyone shaken or push you so hard that you fell? | yes (1); no (0); can't remember (2) | |
|  | Ask question in the questionnaire (**above**) then probe:  1. What is this question asking?  a. Can you repeat the question I asked you in your own words?  2. How did you come to/arrive at the answer that you gave?  a. What did you think about before you gave your answer?  b. How did you go about remembering this?  c. How sure are you about the answer that you gave?  d. How easy or hard was it to give the answer?  3. What influenced you to choose the answer that you gave? | | |
| 8.1.73 | Before you were 18, did anyone ever stab or cut you with a knife or sharp object? | yes (1); no (0); can't remember (2) | |
|  | Ask question in the questionnaire (**above**) then probe:  1. What is this question asking?  a. Can you repeat the question I asked you in your own words?  2. How did you come to/arrive at the answer that you gave?  a. What did you think about before you gave your answer?  b. How did you go about remembering this?  c. How sure are you about the answer that you gave?  d. How easy or hard was it to give the answer?  3. What influenced you to choose the answer that you gave? | | |
| 8.1.84 | Before you were 18, did anyone ever burn you with a cigarette, an iron or burning object? | yes (1); no (0); can't remember (2) | |
|  | Ask question in the questionnaire (**above**) then probe:  1. What is this question asking?  a. Can you repeat the question I asked you in your own words?  2. How did you come to/arrive at the answer that you gave?  a. What did you think about before you gave your answer?  b. How did you go about remembering this?  c. How sure are you about the answer that you gave?  d. How easy or hard was it to give the answer?  3. What influenced you to choose the answer that you gave? | | |
| 8.1.89 | Before you were 18, did an adult ever hurt you so badly that you had to go to the hospital or clinic? | yes (1); no (0); can't remember (2) | |
|  | Ask question in the questionnaire (**above**) then probe:  1. What is this question asking?  a. Can you repeat the question I asked you in your own words?  2. How did you come to/arrive at the answer that you gave?  a. What did you think about before you gave your answer?  b. How did you go about remembering this?  c. How sure are you about the answer that you gave?  d. How easy or hard was it to give the answer?  3. What influenced you to choose the answer that you gave? | | |
| 8.1.92 | In general before you were 18, how often were you physically hurt (beaten, hit or other acts) compared with other children around your age at the time? | Much less than most children (1); A little less than most children (2); About the same as most children (3); A little more than most children (4); Much more than most children (5) | |
|  | Ask question in the questionnaire (**above**) then probe:  1. What is this question asking?  a. Can you repeat the question I asked you in your own words?  2. How did you come to/arrive at the answer that you gave?  a. What did you think about before you gave your answer?  b. How did you go about remembering this?  c. How sure are you about the answer that you gave?  d. How easy or hard was it to give the answer?  3. What influenced you to choose the answer that you gave? | | |
