# Supplementary material for: Conducting Violence Research Across Multiple Family Generations and with Young Children: Findings from a Mixed-Methods Pilot Study in South Africa
Source: Int J Child Maltreat. 2023 Mar 7:1–27. Online ahead of print. doi: 10.1007/s42448-023-00157-w (PMC9988603; doi:10.1007/s42448-023-00157-w)
Supplement: Supplementary file 2 — Supplementary file2 (DOCX 15 KB) [file 42448_2023_157_MOESM2_ESM.docx]

**Supplemental Table B: Management of referred cases to study social worker**

| **Participant group** | **Reason for referral** | **Mandated reporting required?** | **Social worker management of case** |
| --- | --- | --- | --- |
| Child | Bullying at school | Yes | Counselling of child and parent and advice provided on how to engage with the school on the matter. Visits to schools planned after end of data collection to speak about bullying and the impact it has on child mental health. Mandatory referral to DSD. |
| Child | Disability and chronic illness | Yes | Assessment and referral to DSD for further assessment. |
| Child | Emotional abuse | Yes | Psychosocial support for child, parenting skills provided to parents and referral to DSD for further assessment. |
| Child | Exposure to domestic violence/intimate partner violence | Yes | Psychosocial support for child, counselling provided to parent/caregiver, and referral to local shelter or psychologist, as needed. |
| Child | Health issues (e.g., Bilharzia) | No | Referral to local health clinic. |
| Child | Mental health (anxiety, depression, trauma) | No | Psychosocial support for child and referral to local health clinic, as needed. |
| Child | Physical abuse | Yes | Assessment and mandatory referral to DSD, counselling provided to child participant and where possible and safe for the child, discussion of positive parenting techniques with parents. |
| Child | Sexual violence/abuse | Yes | Assessment and mandatory referral to DSD, counselling provided to the child participant. Counselling of a non-offending caregiver around what constitutes sexual abuse and what to do when it is disclosed or witnessed. |
| Adult | Corporal punishment/Hitting of children/ Harsh parenting | No | 1-2 hour positive parenting workshop with parent, discussion of anger management, and coaching around keeping calm. |
| Adult | Current domestic violence/Intimate partner violence | No | Counselling and referral to local government counsellor. |
| Adult | Disability | No | Referral to health clinic for medical assessment and SASSA for disability grant. |
| Adult | Emotional abuse by family member | No | Referral to victim shelter and DSD. |
| Adult | Food insecurity | No | Family assisted with food parcel (n=1), other families referred to SASSA for the Social Relief of Distress (SRD). |
| Adult | Health issues (e.g., HIV, Bilharzia, Tuberculosis) | No | Referral to local health clinic. |
| Adult | Housing issues | No | Contacting of local councillors regarding RDP houses and Department of Housing. |
| Adult | ID document | No | Referral to Department of Home Affairs. |
| Adult | Late birth registration | No | Referral to SASSA for SRD and Department of Home Affairs for birth registration. |
| Adult | Mental health issues (e.g., psychosis) | No | Referral to local psychologist, counselling also provided. |
| Adult | Non-payment of child maintenance | No | Social worker mediated between parents and father restarted payments. |
| Adult | Self-referral | No | Counselling, support, and referrals provided according to need. |
| Adult | Sexual violence | No | Referral to local health clinic if occurred <72 hours ago. Counselling and referral to local psychologist, as needed. |
| Adult | Suicide ideation/Suicidality | No | Counselling and referral to local psychologist. |
